# Supplementary material for: Effect of Fermentation Scale on Microbiota Dynamics and Metabolic Functions for Indigo Reduction
Source: Int J Mol Sci. 2023 Sep 28;24(19):14696. doi: 10.3390/ijms241914696 (PMC10572741; doi:10.3390/ijms241914696)
Supplement: Supplementary file 1 [file ijms-24-14696-s001.zip › Supplementary Figs..pdf]

Effect of Fermentation Scale on Microbiota dynamics and Metabolic Functions for Indigo Reduction

Nowshin Farjana [1,2,†], Zhihao Tu [1,2,†], Hiromitsu Furukawa [3], Hisako Sumi [4], and Isao Yumoto [1,2*]

[1]*Bioproduction Research Institute, National Institute of Advanced Industrial Science and Technology (AIST), Sapporo, Japan*
[2]*Laboratory of Environmental Microbiology, Graduate School of Agriculture, Hokkaido University, Sapporo, Japan*
[3]*North-Indigo Textile Arts Studio, Otaru, Japan*
[4]*Sensing system Research Center, National Institute of Advanced Industrial Science and Technology (AIST), Tsukuba, Japan*

*Correspondence: i.yumoto@aist.go.jp

† These authors contributed equally to this work.

Keywords: alkaliphile; convergence of microbiota; extracellular electron transport

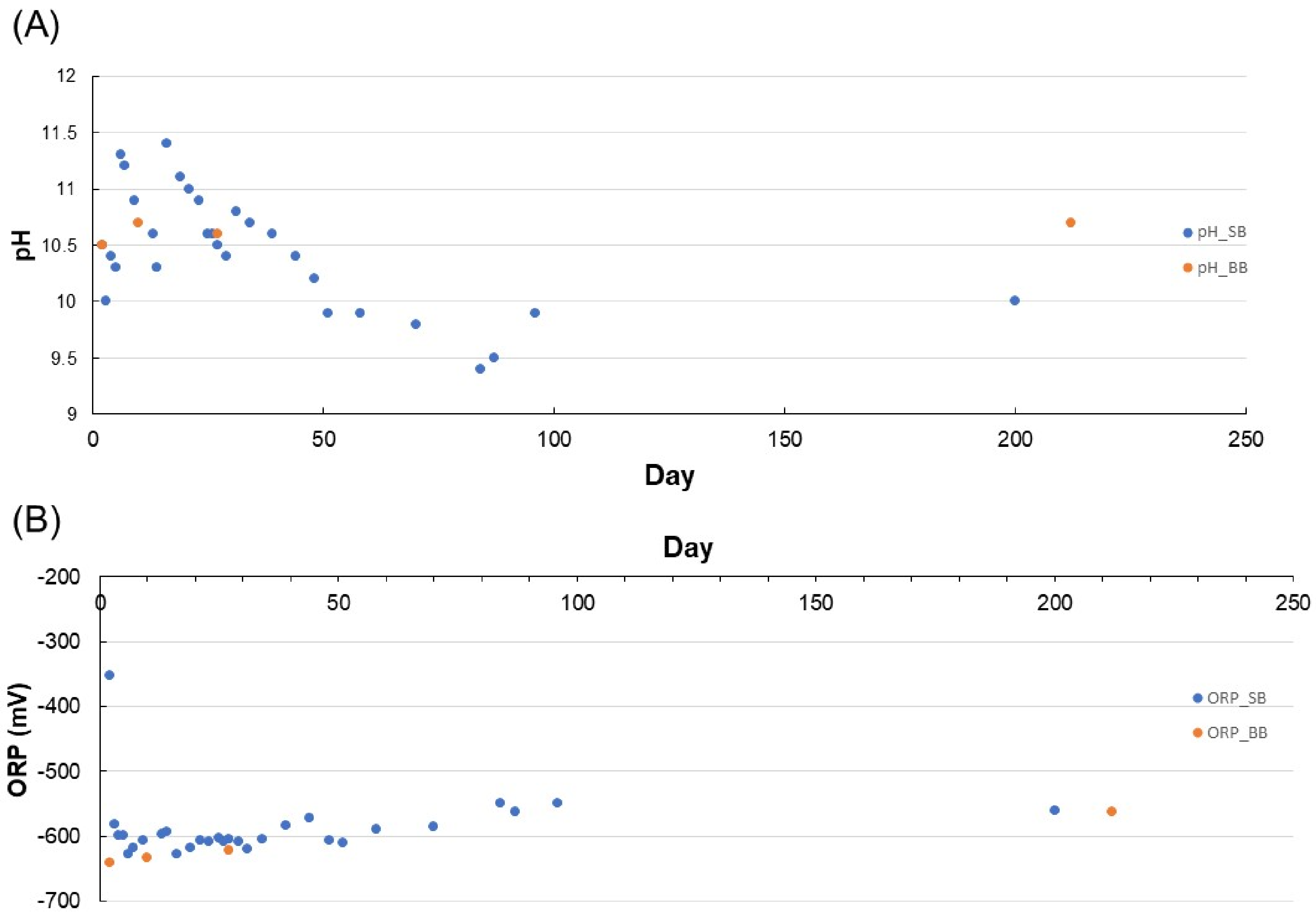
(A)
pH
Day
pH_SB
pH_BB
12
11.5
11
10.5
10
9.5
9
0
50
100
150
200
250
(B)
Day
ORP (mV)
ORP_SB
ORP_BB
-200
-300
-400
-500
-600
-700
0
50
100
150
200
250

Figure S1. Changes in pH (A) and redox potential (ORP) (B) in big- (orange circle) and small-scale (blue circle) batches during indigo fermentation.

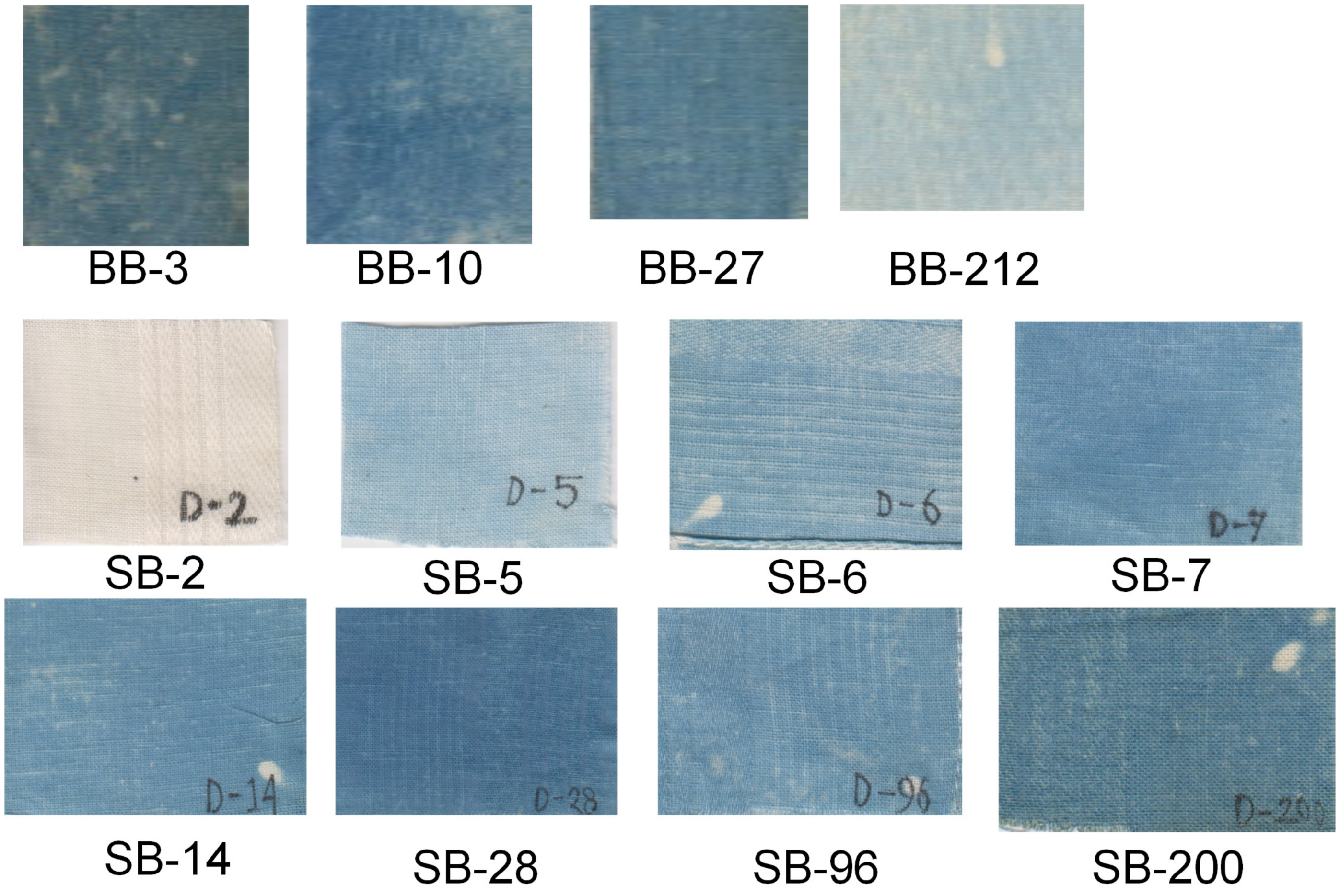

Figure S2. The blue color depth of the dyed cotton cloth shows the indigo reduction state in the big- (BB) and small-scale (SB) batches. The numbers are fermentation days for each batch.

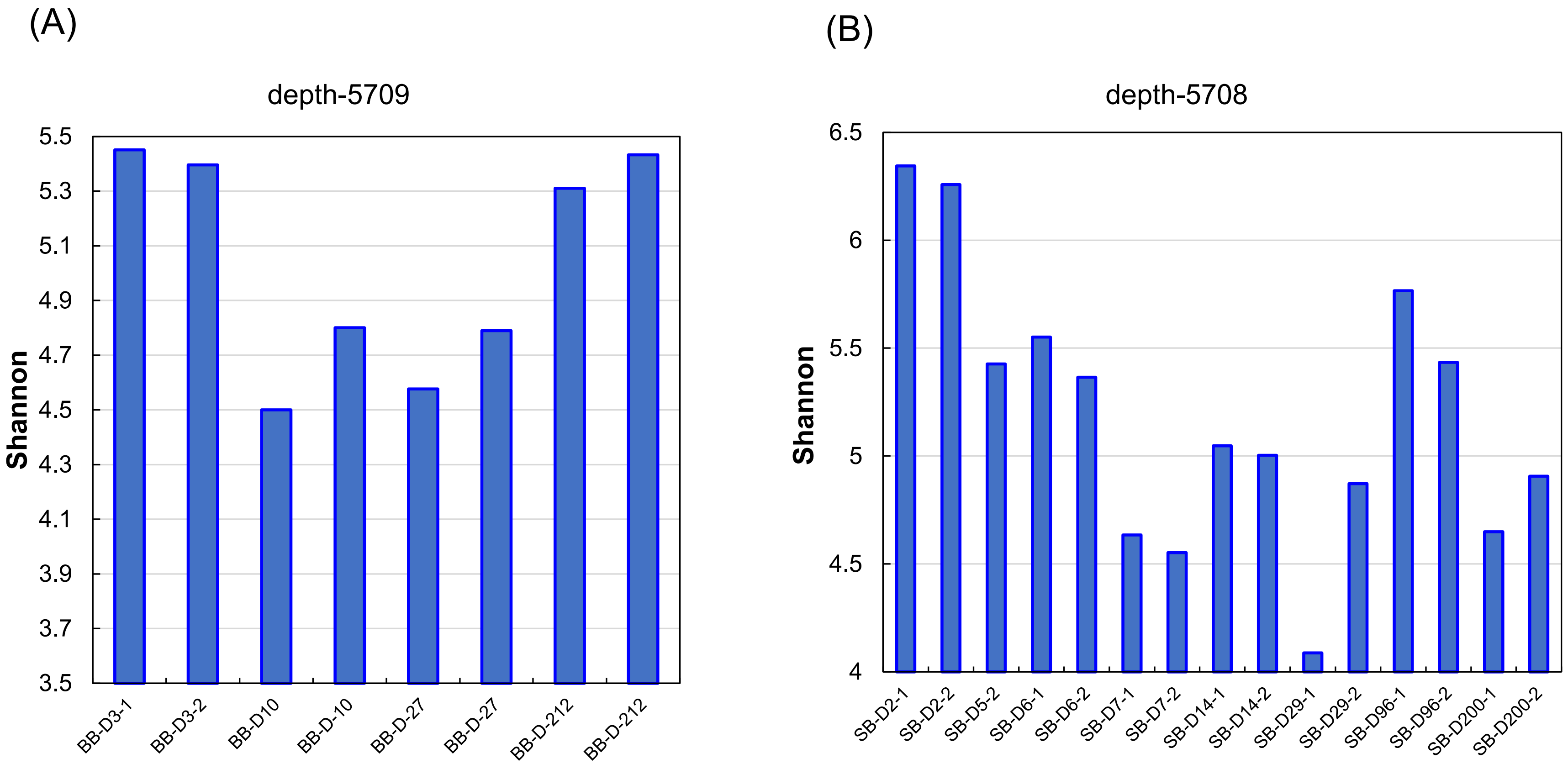

(A)
depth-5709
Shannon
5.5
5.3
5.1
4.9
4.7
4.5
4.3
4.1
3.9
3.7
3.5
BB-D3-1
BB-D3-2
BB-D10
BB-D-10
BB-D-27
BB-D-27
BB-D-212
BB-D-212
(B)
depth-5708
Shannon
6.5
6
5.5
5
4.5
4
SB-D2-1
SB-D2-2
SB-D5-2
SB-D6-1
SB-D6-2
SB-D7-1
SB-D7-2
SB-D14-1
SB-D14-2
SB-D29-1
SB-D29-2
SB-D96-1
SB-D96-2
SB-D200-1
SB-D200-2

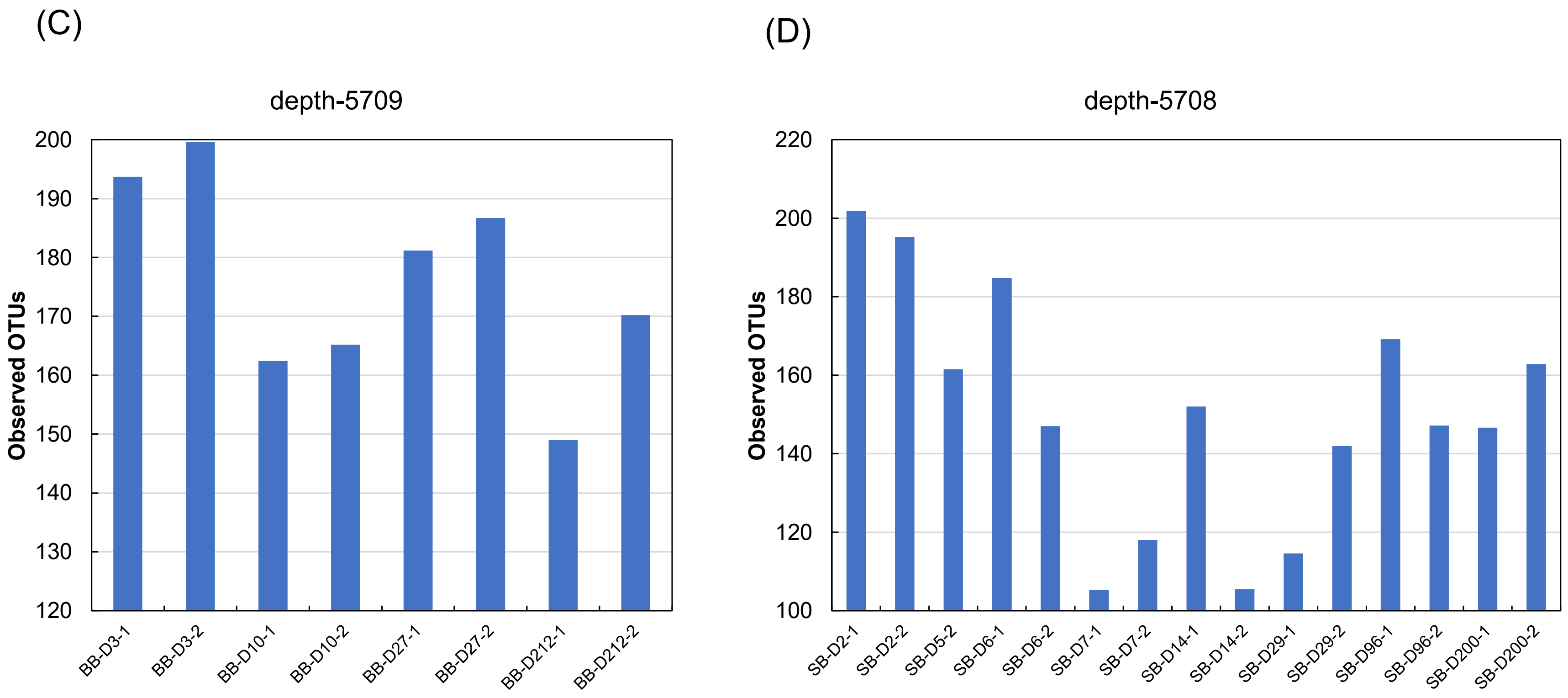

Figure S3. Changes in alpha diversity of big- (A and C) and small-scale (B and D) fermentation batches. The Shannon index (A and B) and observed operational taxonomic units (OTUs) for the different fermentation periods were analyzed using the Divisive Amplicon Denoising Algorithm (DADA2). The 16S rRNA gene sequencing depths were 5709 and 5708 for big- and small-scale batches, respectively. The values represent the average of ten random samplings. The standard deviations were < 0.13 in Shannon index and < 0.95 and < 4.4 in big and small batches, respectively in the observed OTUs.

Category/Superpathway/Subpathway	D3	D10	D27	D212	D10/D3	D27/D3	D10/D212	D27/D212
**Cellular Processes**								
Transport and catabolism								
Prokaryotic defense system	1.42%	1.60%	1.57%	1.10%	1.13	1.10	1.46	1.43
**Genetic Information Processing**								
Replication and repair								
Chromosome and associated proteins	2.43%	2.46%	2.45%	2.28%	1.01	1.01	1.08	1.07
DNA repair and recombination proteins	3.25%	3.27%	3.28%	3.04%	1.01	1.01	1.08	1.08
Translation								
Aminoacyl-tRNA biosynthesis	0.80%	0.82%	0.81%	0.78%	1.02	1.01	1.06	1.04
Ribosome	4.92%	5.04%	4.97%	4.58%	1.02	1.01	1.10	1.09
Translation factors	1.05%	1.07%	1.06%	1.03%	1.02	1.01	1.05	1.03
**Metabolism**								
Amino acid metabolism								
Histidine metabolism	0.92%	0.92%	0.90%	0.85%	1.00	0.98	1.08	1.07
Phenylalanine, tyrosine and tryptophan biosynthesis	1.20%	1.17%	1.19%	1.11%	0.97	0.99	1.05	1.07
Carbohydrate metabolism								
Amino sugar and nucleotide sugar metabolism	1.31%	1.35%	1.36%	1.16%	1.03	1.04	1.16	1.18
Glycolysis / Gluconeogenesis	0.70%	0.71%	0.71%	0.59%	1.01	1.01	1.19	1.19
Pentose phosphate pathway	0.91%	0.91%	0.89%	0.77%	1.00	0.98	1.18	1.16
Pyruvate metabolism	1.00%	1.02%	0.97%	0.85%	1.03	0.98	1.21	1.15
Starch and sucrose metabolism	1.75%	1.82%	1.84%	1.28%	1.04	1.05	1.43	1.44
Glycan biosynthesis and metabolism								
Peptidoglycan biosynthesis	0.73%	0.75%	0.74%	0.67%	1.03	1.02	1.12	1.11
Peptidoglycan biosynthesis and degradation proteins	1.03%	1.08%	1.05%	0.98%	1.05	1.02	1.11	1.08
Lipid metabolism								
Glycerophospholipid metabolism	1.01%	1.02%	0.97%	0.88%	1.02	0.97	1.16	1.10
Metabolism of terpenoids and polyketides								
Terpenoid backbone biosynthesis	0.84%	0.85%	0.82%	0.74%	1.02	0.98	1.14	1.10
**Unclassified**								
Genetic information processing								
Replication, recombination and repair proteins	1.82%	2.04%	1.99%	1.40%	1.12	1.09	1.46	1.42
Metabolism								
Others	0.99%	0.98%	1.00%	0.83%	0.98	1.01	1.18	1.21

1.5
1.2
1.0

Figure S4. Functional abundance (orange bar) and functional abundance ratios between sample with different dye intensities (D10/D3, D27/D3, D10/D212 and D27/D212) in the big batch. Subpathways are marked in the ratios in D10/D212and D27/D212 as follows: > 1.40: yellow; > 1.15: pale blue; and ≥ 1.04: pale green. Metagenomic predictions produced using PICRUSt2 and BURRITO are shown. Subpathways containing a number ≥ 1.05 in either ratio in D10/D212 and D27/D212 were selected. Abbreviations: D, day.

Category/Superpathway	Subpathway	D2	D7	D29	D7/D2	D29/D2	
**Cellular Processes**							
Cell motility	Bacterial motility proteins	1.41%	1.68%	1.54%	1.19	1.09	
Transport and catabolism	Prokaryotic Defense System	0.77%	1.06%	1.43%	1.39	1.87	
**Environmental Information Processing**							
Membrane transport	Phosphotransferase system (PTS)	0.17%	0.40%	0.43%	2.35	2.53	
	Secretion system	1.31%	1.35%	1.44%	1.03	1.10	
**Genetic Information Processing**							
Replication and repair	Chromosome and associated proteins	1.88%	2.19%	2.28%	1.17	1.22	
	DNA repair and recombination proteins	2.85%	3.08%	3.14%	1.08	1.10	
	DNA replication proteins	0.64%	0.75%	0.81%	1.18	1.27	
Transcription	Transcription factors	3.65%	3.89%	3.96%	1.07	1.08	
Translation	Aminoacyl-tRNA biosynthesis	0.68%	0.80%	0.79%	1.19	1.16	
	Ribosome	3.96%	4.83%	4.81%	1.22	1.22	
	Ribosome biogenesis	2.75%	3.39%	3.47%	1.23	1.26	
	Transfer RNA biogenesis	2.72%	3.05%	3.06%	1.12	1.13	
	Translation factors	0.92%	1.06%	1.04%	1.15	1.13	
**Metabolism**							
Amino acid metabolism	Lysine biosynthesis	0.64%	0.67%	0.71%	1.04	1.11	
	Phenylalanine, tyrosine and tryptophan biosynthesis	1.10%	1.26%	1.19%	1.15	1.08	
Carbohydrate metabolism	Amino sugar and nucleotide sugar metabolism	1.05%	1.17%	1.28%	1.12	1.22	
	Glycolysis / Gluconeogenesis	0.54%	0.61%	0.65%	1.13	1.20	
	Starch and sucrose metabolism	0.91%	1.27%	1.62%	1.40	1.78	
Glycan biosynthesis and metabolism	Peptidoglycan biosynthesis	0.54%	0.66%	0.70%	1.23	1.28	
	Peptidoglycan biosynthesis and degradation proteins	0.84%	0.97%	1.00%	1.15	1.18	
Metabolism of cofactors and vitamins	Thiamine metabolism	0.64%	0.72%	0.69%	1.12	1.07	
Metabolism of terpenoids and polyketides	Terpenoid backbone biosynthesis	0.71%	0.81%	0.79%	1.14	1.11	
Nucleotide metabolism	Purine metabolism	2.31%	2.37%	2.48%	1.03	1.07	
	Pyrimidine metabolism	1.58%	1.86%	1.87%	1.18	1.18	2.5
**Unclassified**							2.0
Cellular processes and signaling	Cell growth	2.47%	2.71%	2.58%	1.10	1.05	1.5
Genetic information processing	Replication, recombination and repair proteins	1.07%	1.40%	1.77%	1.31	1.65	1.0
Metabolism	Energy metabolism	1.34%	1.47%	1.49%	1.10	1.11	

Figure S5. Functional abundance (orange bar) and functional abundance ratio between different dyeing intensity samples (D7/D2 and D29/D2) in the small batch. Subpathways are marked in the average ratio in D7/D2 and D29/D2 as follows: > 1.40: yellow; > 1.15: pale blue; ≥ 1.05: pale green. Metagenomic predictions produced using PICRUSt2 and BURRITO are shown. Subpathways containing a number ≥ 1.05 in either ratio in D7/D2 and D29/D2 were selected. Abbreviations: D, day.

Category/Superpathway	Subpathway	D29	D96	D200	D29/D200	D29/D96	
**Cellular Processes**							
Transport and catabolism	Prokaryotic Defense System	1.43%	0.87%	0.97%	1.49	1.64	
**Environmental Information Processing**							
Membrane transport	Phosphotransferase system (PTS)	0.43%	0.24%	0.22%	1.92	1.77	
**Genetic Information Processing**							
Replication and repair	Chromosome and associated proteins	2.28%	2.06%	2.15%	1.06	1.11	
	DNA repair and recombination proteins	3.14%	2.92%	2.86%	1.10	1.07	
	DNA replication proteins	0.81%	0.73%	0.74%	1.10	1.12	
Transcription	Transcription factors	3.96%	3.24%	3.17%	1.25	1.22	
	Transcription machinery	1.37%	1.31%	1.29%	1.06	1.05	
Translation	Aminoacyl-tRNA biosynthesis	0.79%	0.73%	0.70%	1.13	1.07	
	Ribosome	4.81%	4.32%	4.10%	1.17	1.11	
**Metabolism**							
Amino acid metabolism	Cysteine and methionine metabolism	1.02%	0.96%	0.87%	1.17	1.06	
	Lysine biosynthesis	0.71%	0.67%	0.65%	1.10	1.06	
	Phenylalanine, tyrosine and tryptophan biosynthesis	1.19%	1.06%	1.00%	1.19	1.12	
Carbohydrate metabolism	Amino sugar and nucleotide sugar metabolism	1.28%	1.03%	1.00%	1.29	1.25	
	Fructose and mannose metabolism	0.46%	0.36%	0.28%	1.68	1.30	
	Glycolysis / Gluconeogenesis	0.65%	0.50%	0.48%	1.34	1.30	
	Pentose phosphate pathway	0.85%	0.64%	0.61%	1.37	1.32	
	Starch and sucrose metabolism	1.62%	0.75%	0.77%	2.11	2.15	
Enzyme families	Peptidases	2.54%	2.15%	2.12%	1.20	1.18	
Glycan biosynthesis and metabolism	Peptidoglycan biosynthesis	0.70%	0.53%	0.51%	1.37	1.30	
Metabolism of cofactors and vitamins	Thiamine metabolism	0.69%	0.65%	0.61%	1.12	1.06	
Metabolism of terpenoids and polyketides	Terpenoid backbone biosynthesis	0.79%	0.71%	0.66%	1.18	1.11	
Nucleotide metabolism	Purine metabolism	2.48%	2.39%	2.26%	1.10	1.04	
	Pyrimidine metabolism	1.87%	1.62%	1.57%	1.19	1.15	
**Unclassified**							2.5
Cellular processes and signaling	Cell growth	2.58%	0.98%	0.97%	2.67	2.64	2.0
Genetic information processing	Replication, recombination and repair proteins	1.77%	1.37%	1.53%	1.16	1.30	1.5
Metabolism	Carbohydrate metabolism	0.59%	0.37%	0.32%	1.82	1.61	1.0
	Others	0.90%	0.91%	0.81%	1.11	0.99	

Figure S6. Functional abundance (orange bar) and functional abundance ratio between sample with different dye intensities (D29/D200 and D29/D96) in the small batch. Subpathways are marked in the average ratio in D29/D200 and D29/D96 as follows: > 1.50: yellow; > 1.30: cream; ≥ 1.15: pale blue; ≥ 1.05: pale green. Metagenomic predictions produced using PICRUSt2 and BURRITO are shown. Subpathways containing a number ≥ 1.05 in either ratio in D10/D121 and D27/D122 were selected. Abbreviations: D, day.
